# Metastasis of serous ovarian carcinoma to the breast: a case report and review of the literature

**DOI:** 10.1186/s13256-024-04445-y

**Published:** 2024-03-26

**Authors:** Sofia Dueño, Rachel Stein, Mohsin Jamal, Gregory Lewis, Karina Hew

**Affiliations:** 1 Department of Obstetrics and Gynecology, University of Florida College of Medicine, Jacksonville, FL USA; 2 Department of Radiology, University of Florida College of Medicine, Jacksonville, FL USA; 3 Department of Molecular and Digital Pathology, University of Florida College of Medicine, Jacksonville, FL USA; 4 Division of Minimally Invasive Gynecologic Surgery, Department of Obstetrics and Gynecology, University of Florida College of Medicine, Jacksonville, FL USA; 5 Division of Gynecology Oncology, Department of Obstetrics and Gynecology, University of Florida College of Medicine-Jacksonville, 653-1 West 8th Street, Jacksonville, FL 32209 USA

**Keywords:** Ovarian cancer, Breast metastasis, Ovarian adenoarcinoma

## Abstract

**Background:**

Breast metastasis from primary ovarian cancer is rare, with an estimated frequency of 0.07%. More than 110 cases are reported in the literature of metastatic spread of ovarian cancer to the breast and axilla. This entity usually represents aggressive late disease characterized by multi-drug chemoresistance and a poor prognosis with a median survival time of 16 months. Currently no standardized treatment protocol exists for this condition.

**Case presentation:**

We present a case of a 59-year-old Caucasian female with recurrent high-grade serous ovarian cancer who was diagnosed with symptomatic unilateral breast metastasis while on fourth line chemotherapy with weekly paclitaxel. She was treated with local radiation with 2300 cGy to the right breast with a complete response. She then had a subsequent recurrence in the ipsilateral breast 8 months after completion of post treatment imaging. She remains alive to date approximately 2 years after her initial diagnosis of breast metastasis on seventh line treatment.

**Conclusions:**

Breast metastasis from primary ovarian cancer is rare and represents advanced disease characterized by multi-drug chemoresistance and a poor prognosis. This case describes radiation therapy as a safe, effective treatment option to improve local control and quality of life in these patients, but with limited durability of response.

## Background

Ovarian cancer is the fifth leading cause of cancer deaths in women in the United States. It is characterized by advanced presentation with metastasis confined to the peritoneal cavity in 85% of patients [1]. Metastasis to distant sites via hematologic and lymphatic spread can also occur but is uncommon. Spread to the breast from serous ovarian cancer is a rare occurrence with a limited body of literature reporting these instances. The estimated frequency of serous ovarian cancer metastasis to the breast is 0.07% [2]. These cases are representative of aggressive, advanced disease characterized by chemoresistance and a poor prognosis with a median survival time of 16 months [3]. However, given the rarity of this condition, there is no standardized treatment protocol at this time. Herein we present the case of a patient with unilateral breast metastasis from high-grade serous ovarian cancer treated with local radiation and subsequent recurrence of breast metastasis from high-grade serous ovarian cancer. This case report serves as an addition to the growing literature on ovarian cancer metastasis to rare, distant sites and provides a distinct example of recurrence of breast metastasis from high-grade serous ovarian cancer after local radiation therapy. This case also provides a brief review of existing literature detailing current modalities of treatment and prognosis following this diagnosis.

## Case presentation

This is a 59-year-nulligravid Caucasian female with recurrent platinum resistant high-grade serous ovarian adenocarcinoma of the ovary. Her medical history was significant for hyperlipidemia, hypertension and hypothyroidism. Her family history was significant for a paternal grandfather with colon cancer but otherwise negative for uterine, ovarian or breast cancer. She was a former smoker (1/2 packs per day, quit over 10 years ago), she denied alcohol abuse or illicit drug use.

She initially presented with symptoms of left lower quadrant pain, bloating, and constipation. Her diagnostic evaluation included CT imaging suggestive of a left ovarian mass and widely metastatic disease in the abdomen and pelvis, a colonoscopy showing extrinsic compression and stricture of the rectum, and an elevated CA − 125 of 673. The diagnosis of stage IIIC high grade serous ovarian adenocarcinoma was confirmed with surgical biopsies taken at the time of diagnostic laparoscopy. She subsequently underwent four cycles of neoadjuvant chemotherapy with carboplatin and paclitaxel followed by an optimal interval cytoreductive surgery. Surgery included a modified posterior exenteration, omentectomy, radical pelvic dissection, and a loop ileostomy and no gross residual disease was identified. Surgery was followed by three additional cycles of adjuvant chemotherapy and maintenance therapy with niraparib. Of note, she underwent genetic testing which confirmed her negative BRCA 1/2 status. She also had tumor molecular profiling which showed positive biomarker expression for homologous recombination deficiency (HRD), programmed death 1 ligand (PDL-1), and folate receptor alpha (FRα). These results were used in accordance with NCCN guidelines to determine her initial and subsequent treatment options.

**Fig. 1:**
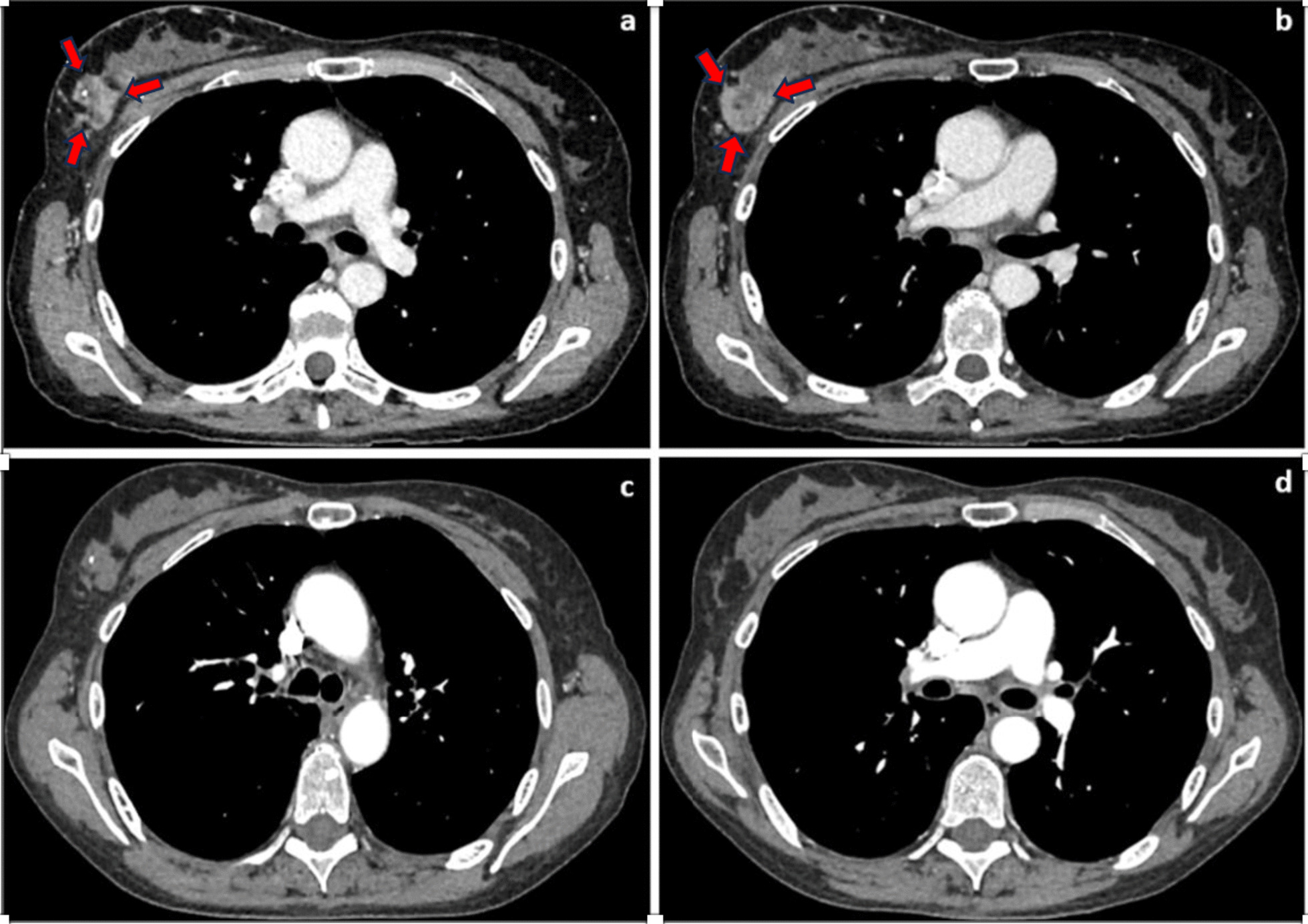
Axial CT chest with intravenous contrast. Pre-treatment CT chest (**a**, **b**) demonstrates a heterogenous enhancing mass (red arrows) in the right upper outer breast. Biopsy clip is noted. Post-treatment CT chest (**c**, **d**) demonstrates no enhancement and decreased size of the right upper outer breast mass, particularly the more inferior portion (**d**)

**Fig. 2:**
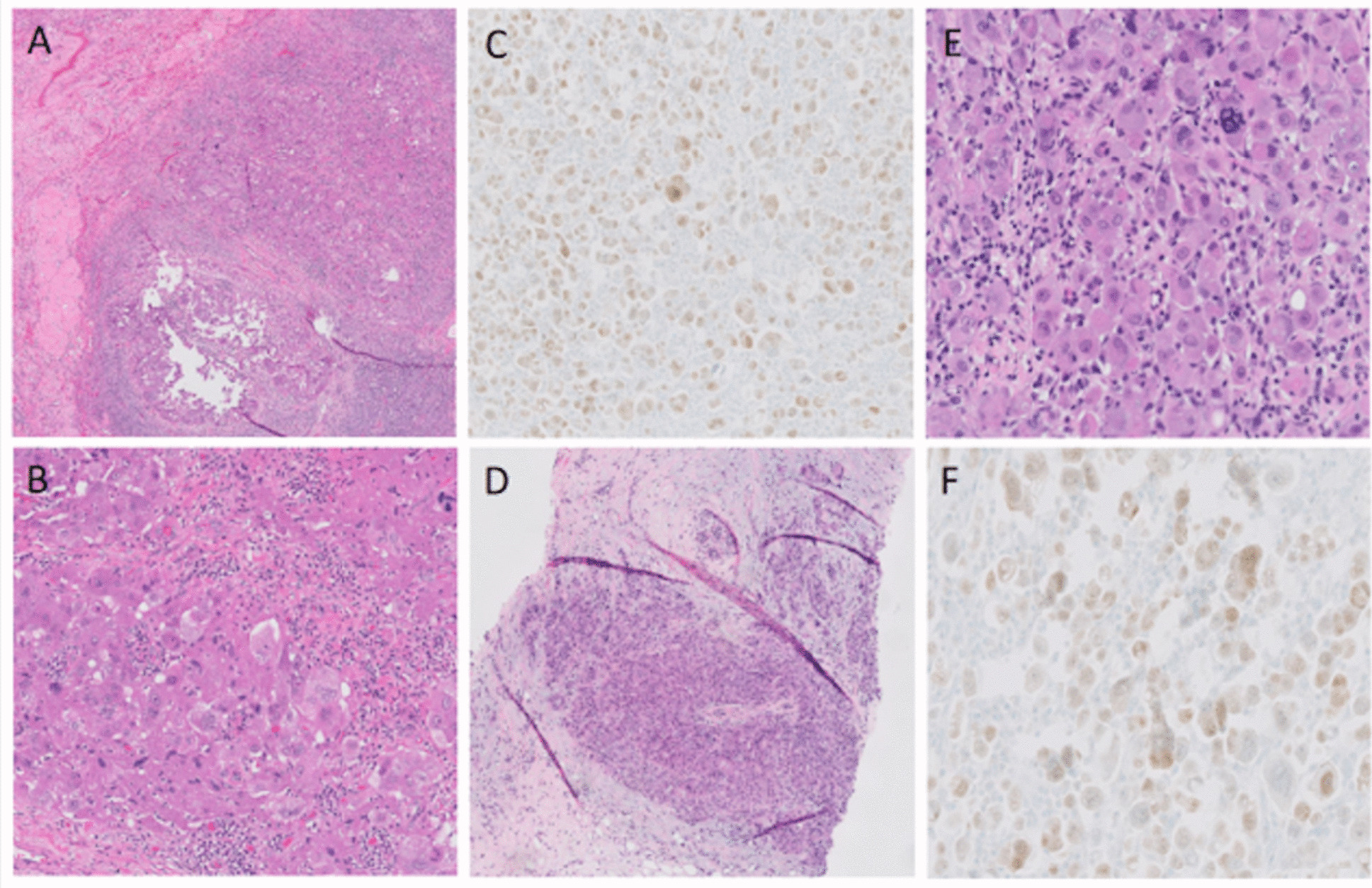
Histopathological sections and immunochemistry demonstrating metastatic ovarian high-grade serous carcinoma. **A** Histologic sections of the left ovary with infiltrative tumor cells in the ovarian stroma (arrowhead, H&E stain at 5x). **B** On higher magnification, the tumor cells are large with hyperchromatic nuclei (arrowhead, H&E stain at 20x). **C** The tumor cells are immunoreactive for PAX-8 (immunohistochemical stain with a brown chromogen, 20x). **D** Histologic sections of the right breast with infiltrative tumor cells (arrowhead, H&E stain at 5x). **E** On higher magnification, the tumor cells are morphologically similar to the patient’s ovarian tumor (arrowhead, H&E stain at 20x). **F** PAX-8 immunohistochemical stain with brown chromogen highlights the tumor cells, confirming the diagnosis of metastatic ovarian high-grade serous carcinoma (20x)

She had a disease-free interval of 8 months following her initial diagnosis followed by wide metastatic recurrence to the abdomen, chest, and cervical lymph nodes and was treated with multiple lines of chemotherapeutic agents and targeted therapies. Approximately 24 months from her original diagnosis and during her fourth line of treatment with weekly paclitaxel, she presented with complaints of a painful mass in her right breast. On clinical evaluation she was found to have a palpable, tender, firm, and irregular 5.5-cm right breast mass in the upper-outer quadrant with nipple retraction and right axillary lymphadenopathy. This mass was also demonstrated on interval CT imaging (Fig. 1a, b) and was confirmed with a diagnostic mammogram and ultrasound of the right breast. She underwent ultrasound guided biopsies of the right breast mass and axillary lymph nodes which confirmed metastatic high grade serous ovarian adenocarcinoma (Fig. 2A–F).

She subsequently received palliative radiation to the right breast using the Baillet regimen. A total dose of 2300 cGy was delivered in 4 fractions: 500 cGy on days 1 and 3 and 650 cGy on days 15 and 17. After completion of radiation the patient was started on her fifth line of chemotherapy with pembrolizumab and olaparib. She had an excellent clinical and radiologic response to treatment as noted on physical exam and 12-week post-treatment imaging (Fig. 1c, d) with no significant treatment related toxicity. The patient presented 8 months after post-treatment imaging with a new right breast mass in the previously radiated field and worsening lymphadenopathy. She is currently on a seventh line of chemotherapy with mirvetuximab-soravtansine, gynx which is a folate receptor alpha (FRα)-directed antibody and microtubule inhibitor conjugate. She remains alive to date with stable disease approximately 2 years after the breast metastasis was initially diagnosed.

## Discussion

There are over 110 cases of ovarian cancer metastasis to the breast. The treatment options include palliative radiation, mastectomy, lumpectomy, and systemic chemotherapy. However, there is no consensus on the optimal treatment protocol for this condition. On review of the literature, these cases are primarily treated with systemic chemotherapy in combination with local radiation therapy. Surgical interventions are mostly reserved for patients who are unresponsive to systemic chemotherapy and opt for palliation treatment [2]. In a retrospective review of 18 cases of ovarian or peritoneal serous carcinoma with metastasis to breast/axillary lymph nodes, all patients underwent varying forms of chemotherapy with one patient additionally undergoing mastectomy with axillary lymph node dissection after initially being diagnosed with infiltrating ductal carcinoma [1]. The table provided below summarizes current cases in the existing literature that describe the treatment, time to recurrence, and prognosis of ovarian cancer patients with breast metastases (Table 1). 8 cases implemented systemic chemotherapy in their initial treatment of metastatic ovarian carcinoma to the breast, and one case added adjunctive local radiation to their treatment regimen [8]. Three cases included surgical management in their initial treatment, with two of those cases having surgical management before initiation of chemotherapy for suspected primary breast cancer [11, 12] and the other case performing surgery for palliative treatment [4]. One case used surgery as a diagnostic tool to confirm metastasis to the breast rather than for treatment [11].

**Table 1:** Previous case reports of serous ovarian carcinoma with metastasis to the breast

Author (year) [ref.]	Age at diagnosis	Time to breast metastasis diagnosis	Treatment of breast metastasis	Breast metastasis recurrence	Time to metastasis recurrence	Treatment of metastasis recurrence	Survival time after breast metastasis
Chiva *et al*. 2022 [4]	79	7 years	Chemotherapy Palliative radical mastectomy				2 months (death)
Musa *et al*. 2022 [5]	Unknown	5 years	Local radiation (5200 cGy)	Yes	1 month	Local radiation (5200 cGy) Chemotherapy (in contralateral breast)	> 2 months (ATP)*
Maeshima *et al*. 2021 [6]	69	9 years	Chemotherapy				24 months (ATP)
Caruso *et al*. 2020 [7]	53	2 years	Chemotherapy	Yes	2 months	Local radiation (3500 Gy) Chemotherapy	36 months (death)
Abu-Tineh *et al*. [8]	54	14 months	Chemotherapy Local radiation (3000 cGy)				24 months (ATP)
Wang *et al*. 2020 [9]	40	6 years	Chemotherapy				4 months (LTF)**
Antuono *et al*. 2018 [10]	54	3 years	Chemotherapy				5 months (ATP)
Tempfer *et al*. 2016 [11]	51	1 year	Chemotherapy Mastectomy				6 months (LTF)
Cormio *et al*. 2001 [12]	43	3 months	Chemotherapy Radical mastectomy				14 months (death)
Amichetti *et al*. 1990 [13]	50	10 months	Excision Local radiation				8 months (death)

*ATP: Alive at time of publication of case report

**Lost to follow up

While most of the reviewed cases revealed either progressive or improved metastatic disease, two of the reports had documented recurrence of ovarian cancer metastasis to the breast [5, 7] This is also a specific finding in the patient of this case report as she had ipsilateral recurrent right ovarian cancer metastasis after local radiation treatment and subsequent radiologic imaging that showed improvement in disease in the breast. Almost all reported cases in the literature of metastatic ovarian cancer to the breast have been indicative of advanced multi-drug resistant disease and this characteristic can be especially seen in the reported cases of recurrence where patients received multiple lines of systemic chemotherapy after having recurrent metastasis to the breast. In addition to having advanced chemoresistance, cases with ovarian cancer metastasis to the breast carry an overall poor prognosis. Survival ranges from 13 days to 3.5 years, with many patients dying within the first year from the diagnosis of breast metastasis [10]. A retrospective cohort of 169 patients from a single university cancer center in the United States with pathology confirmed metastasis to the breast from extramammary primary solid organ tumors revealed a 10-month median duration of survival from the time of breast metastasis diagnosis [13]. In a case series over a 14-year period (1990–2003) at a university cancer center in the United States of 18 patients with ovarian or peritoneal serous ovarian carcinoma with metastasis to the breast and/or axillary lymph nodes, seven patients died of disease after an averaged 12-month follow-up period [1]. Current literature demonstrates the poor survival outcomes for patients with metastatic high-grade ovarian cancer to the breast, our patient has exceeded the mean survival time of 16 months and is still alive 2 years since her diagnosis of breast metastasis.

While there is no consensus regarding a specific treatment regimen for ovarian cancer metastasis to the breast, chemotherapy was the most common treatment modality used in the treatment of breast metastasis and only one case used concurrent local radiation [8]. Only a small subset of these case reports in the literature includes the use of radiotherapy as the primary treatment of breast metastasis. Local radiation was chosen for this patient because her breast metastasis was chemoresistant and it could provide rapid tumor regression for symptom control. This was then followed by resumption of systemic chemotherapy for control of other metastatic sites. While there is no consensus on optimal treatment of breast cancer metastasis from ovarian cancer, our case demonstrates that radiation can be an effective tool in managing chemoresistant disease as the breast mass completely resolved after treatment. However, the durability of this response remains in question as a new breast mass was identified in the previously radiated field only eight months after her post treatment imaging showing complete resolution. It is unclear if the durability of her response was impacted by the radiation protocol chosen as the total dosage administered was lower than the other regimens noted in our literature review.

One identifiable strength of this case report includes the longevity and follow up time for this patient, which is 2 years after her breast metastasis was diagnosed. She also demonstrated a positive response to local breast radiation while on much later line of treatment compared to other cases described in literature. In most of the other cases described, breast metastasis occurred while on earlier lines of therapy or in patients who had expired shortly after treatment with limited follow up. This patient’s extended survival could be attributed to her tumor biology, younger age, and excellent baseline performance status which allowed her to undergo an extensive cytoreductive surgery which impacts overall survival. These patient factors may have also impacted her ability tolerate multiple lines of therapy. One notable weakness is that the radiation regimen used for treatment was selected based on our institution’s practice pattern and,as previously stated, was a lower total dose compared to other regimens described in the literature.

## Conclusion

Metastatic spread of ovarian cancer to the breast is a rare occurrence associated with chemotherapy-resistance and a poor prognosis. Currently, there are no established guidelines regarding standardized treatment in this clinical scenario. However, our manuscript adds to the existing literature that radiation therapy is a feasible option for local control of metastatic disease to the breast with minimal toxicity and an excellent treatment response which positively impacts quality of life. However, heterogeneity of radiation regimens may impact efficacy and durability of response. Given these findings, more studies are needed to develop a standardized radiation protocol that can be used as a part of a multimodal approach when treating this condition.

## Data Availability

All data generated or analyzed during this case report are included in this published article and its supplementary information.
